# Implementation of a Personalized Risk Model for Lymph Node Metastasis in Endometrial Carcinoma: Healthcare Providers' Perspectives on Use, Barriers, and Facilitators

**DOI:** 10.1002/cam4.71103

**Published:** 2025-08-01

**Authors:** Marike S. Lombaers, Stephanie W. Vrede, Casper Reijnen, Dorry Boll, Nicole C. M. Visser, Johanna M. A. Pijnenborg, Nicole P. M. Ezendam, Rosella P. M. G. Hermens

**Affiliations:** ^1^ Department of Obstetrics and Gynecology Radboud University Medical Center Nijmegen the Netherlands; ^2^ Department of Radiation Oncology Radboud University Medical Center Nijmegen the Netherlands; ^3^ Department of Obstetrics and Gynecology Catharina Hospital Eindhoven the Netherlands; ^4^ Department of Pathology Eurofins PAMM Eindhoven the Netherlands; ^5^ Netherlands Comprehensive Cancer Organization Eindhoven the Netherlands; ^6^ Scientific Center for Quality of Health care (IQ Health) Radboud University Medical Center Nijmegen the Netherlands

**Keywords:** artificial intelligence, endometrial cancer, healthcare providers, lymph node metastases, preoperative counseling, risk estimation model

## Abstract

**Background:**

The ENDORISK model estimates the risk of lymph node metastases (LNM) in endometrial carcinoma (EC) patients using preoperative clinical variables and biomarkers. This qualitative study investigated healthcare providers' (HCP) perspectives on the use of the model and barriers and facilitators for clinical implementation.

**Methods:**

Eight focus group interviews were performed among HCPs. A semi‐structured interview guide was used based on the Grol and Wensing implementation model.

**Results:**

Focus groups included gynecologists, residents of gynecology, pathologists, radiation oncologists, and a nurse specialist (*n* = 41). ENDORISK was deemed supportive for counseling of patients and shared decision‐making for optimal surgical and adjuvant treatment. Barriers for implementation were difficulty in explaining the model and risk percentages to patients, differences in preoperative diagnostic tools used per hospital, and use of the model with the sentinel node procedure. Facilitators were a clear guideline for using the model with a predefined risk cutoff and making the model easily understandable for patients. A 10% risk cutoff was considered clinically relevant for lymph node assessment.

**Conclusion:**

HCP found ENDORISK use in clinical practice supportive for patient counseling. Future implementation should focus on a user‐friendly interface, a cohesive guideline, and training to aid efficient use and counseling of patients.

## 1 Introduction

Endometrial cancer (EC) is the most common malignancy within gynecological oncology. Patients diagnosed with early stage and low‐grade EC have a generally favorable prognosis with a 5‐year survival rate of 73%–83% [1]. However, for those with advanced stage and/or high‐grade EC, the outcome is substantially worse, with 5‐year survival rates between 40% and 58% [2, 3, 4].

Primary treatment for EC is hysterectomy and bilateral salpingo‐oophorectomy. According to international guidelines, surgical lymph node (LN) assessment can be considered in low‐risk patients, but is recommended in high‐risk patients [3, 5]. The risk of lymph node metastases (LNM) is strongly related to tumor grade, myometrial invasion (MI), lymphovascular space invasion (LVSI), and molecular classification [3]. Most of these risk factors, including FIGO stage and LVSI, are not available in a preoperative setting. Expression of simple biomarkers in endometrial biopsies such as estrogen receptor (ER), progesterone receptor (PR), p53, and L1 cell adhesion molecule (L1CAM) shows promising results for predicting LNM, but has so far not been incorporated in guidelines [3, 6, 7, 8]. Optimizing preoperative risk assessment to determine which patients benefit from surgical LN assessment remains relevant even in the era of molecular classification as tumor stage remains the most important prognosticator in the updated FIGO 2023.

With the introduction of the sentinel lymph node (SLN) procedure, the risk of lymphedema related to full lymphadenectomy has reduced significantly [3, 9, 10, 11, 12, 13]. Yet, failure of bilateral SLN mapping occurs in 20%–25%, especially in patients with high body mass index (BMI) (> 30 kg/m^2^) or nonendometrioid histology, who still require side‐specific lymphadenectomy [14, 15, 16, 17, 18, 19]. Furthermore, the first randomized controlled trial researching the additional value of the SLN procedure compared to no LN assessment is still ongoing [20]. Especially for the low‐risk group, in which the incidence of LNM is around 5%–10%, this could still mean that the remaining 90% is overtreated [17, 21]. Therefore, additional tools such as the ENDORISK model might be beneficial to preoperatively stratify patients at risk of LNM and determine whether or not side‐specific lymphadenectomy is needed when SLN failed [22].

The ENDORISK model is a clinical decision tool that has been developed with an accurate preoperative risk estimation of LNM [22]. It incorporates biomarkers such as ER, PR, p53, and L1CAM, and other clinical markers, such as preoperative tumor grade and imaging results on lymphadenopathy [22]. External validation in three independent cohorts resulted in a diagnostic accuracy with an area under the curve (AUC) of 0.81 for estimating risk of LNM compared to 0.62 if based on tumor grade only [22, 23, 24]. ENDORISK is a Bayesian Network, which is a graphic representation of the interactions between the incorporated variables, reflecting the underlying carcinogenic pathophysiology of EC. Bayesian Networks are able to handle missing variables, which makes them very suitable for clinical use [22]. With these characteristics, ENDORISK might be a valuable aid to preoperative risk stratification. However, limited literature exists about the implementation of Bayesian Networks or other preoperative risk models for oncological care, and specifically EC, in clinical practice [25, 26, 27]. In an era in which artificial intelligence is being developed at an increasing rate for a wide array of applications in health care, it remains especially important to keep in mind the demands and benefits to the prospective end user: in the case of the ENDORISK model, this is the healthcare provider (HCP) [28]. Knowledge on the perspectives of end users of risk models is needed for successful implementation [29, 30]. Therefore, we conducted a qualitative study among HCPs working with patients with EC to assess barriers and facilitators for: 1. model usage for estimating risk of LNM 2. implementation into clinical practice.

## 2 Materials and Methods

### 2.1 Study Design

Eight focus groups interviews among HCPs involved in EC care were performed between 2019 and 2022 in the Netherlands and reported according to the consolidated criteria for reporting qualitative research (COREQ) checklist (Data S1) [31].

### 2.2 Setting

Gynecologic oncologic care in the Netherlands is organized in eight clinical oncology regions. These include one referral center and several general hospitals. All EC patients are discussed in multidisciplinary tumor boards. Low‐risk patients are treated in general hospitals, whereas patients who require surgical staging are referred to referral centers.

### 2.3 Participants

**FIGURE 1:**
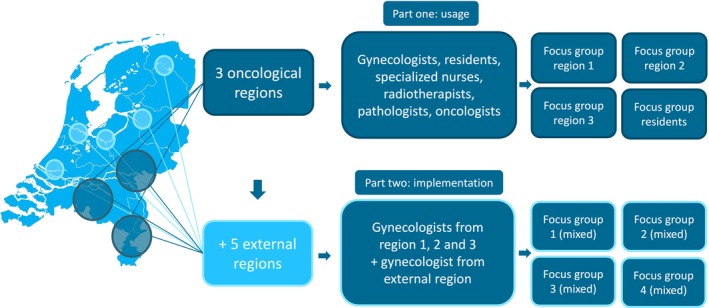
Participant recruitment.

Participant recruitment is visualized in Figure 1. HCPs were invited by e‐mail from three clinical oncology regions in the Netherlands (21 hospitals) by one of the three interviewers (CR, SV, and ML). For part one, a multidisciplinary focus group interview was held per oncological region for which gynecologists, pathologists, radiation oncologists, and nurse specialists were invited. A separate focus group was performed with gynecology residents as they worked in multiple hospitals belonging to different regions. Part 2 included gynecologists, the first anticipated users of the ENDORISK model. Focus groups were no longer stratified by region, and several gynecologists from external regions were approached as well to create a national representative population. The group size aimed for was 6–10 participants [32, 33].

All focus groups had a nonclinical observer present (RH or NE, senior researchers in qualitative research and implementation) and a senior doctor as an independent expert (gynecologist or radiation oncologist). In part one, the clinical initiator of the ENDORISK project was also present (JP, gynecologic oncologist). All focus group interviews were conducted by medical doctors (not gynecologists) and PhD students trained in qualitative research but not working in clinical practice at the time of the focus groups who had extensive knowledge about the ENDORISK model. Due to the COVID‐19 epidemic, three focus groups were face to face and five via Microsoft Teams.

### 2.4 Data Collection

The implementation model of Grol and Wensing was used to formulate interview questions categorized by their implementation domains: innovation, professional, patient, social context, organization, and economical and political context [34]. After the first set of focus groups, the interview guide was updated by changing the general question of each domain of Grol and Wensing from “What are barriers and facilitators for the use of the model within [insert domain]?” to “What are barriers and facilitators for the implementation of the model within [insert domain]?” A translated version of the Dutch interview guide is shown in Data S2.

Before participating in the focus group interviews, participants received an information sheet and signed an informed consent form for participation. Each interview started with a short presentation about the ENDORISK model to explain the different variables included in the model and to show its risk estimation abilities: risk of LNM and chance of survival at 1, 3, and 5 years after diagnosis based on results from the original and two external validation studies [22, 23, 24]. All focus groups were audio‐recorded, and at the end of each focus group, conclusions were summarized and reiterated to participants for feedback.

### 2.5 Analysis

All focus groups were transcribed and anonymized by an external transcription service. The transcripts were independently and iteratively coded with Atlas.ti version 22 by ML and SV, first by deductive reasoning and categorization according to the six domains of the Grol and Wensing model. Inductive reasoning was used for codes that did not fit within the model. Thematic analysis was used to further categorize codes by “use” and “implementation” subcategorized into “facilitator” and “barrier,” to reflect the two aims of the study. After coding, consensus meetings were held by ML, SV, RH, and NE to discuss discrepancies in coding to decide on the definitive code.

Quotes in the results were coded by using “M” for male and “F” for female for the gender of the respondent belonging to the quote. G(number) shows the focus group in which the quote was said.

## 3 Results

### 3.1 Participants

Among the total of 72 invited HCP for all focus groups, 41 (57%) agreed to participate, including gynecologists (*n* = 26), residents of gynecology (*n* = 3), pathologists (*n* = 3), radiation oncologists (*n* = 6), and a nurse specialist. Three gynecologists participated in both the first and second set of focus groups. Distribution and characteristics of participants are shown in Table 1.

**TABLE 1:** Baseline characteristics of healthcare providers participating in the first and second set of focus groups.

Characteristics for Part 1						Characteristics for Part 2
	Gynecologist	Resident Gynecology	Pathologist	Radiation oncologist	Nurse specialist	Gynecologist
Gender						
Male	4	1	1	1		5
Female	7	2	2	5	1	13
Years of experience (mean)	13	—	6	23	2	12
Type of hospital						
Academic	3	3	2	2		5
Referral	3			1	1	2
General	5		1	2		11
Enrollment rate	11/24	3/6	3/6	5/6	1/2	18/29

**FIGURE 2:**
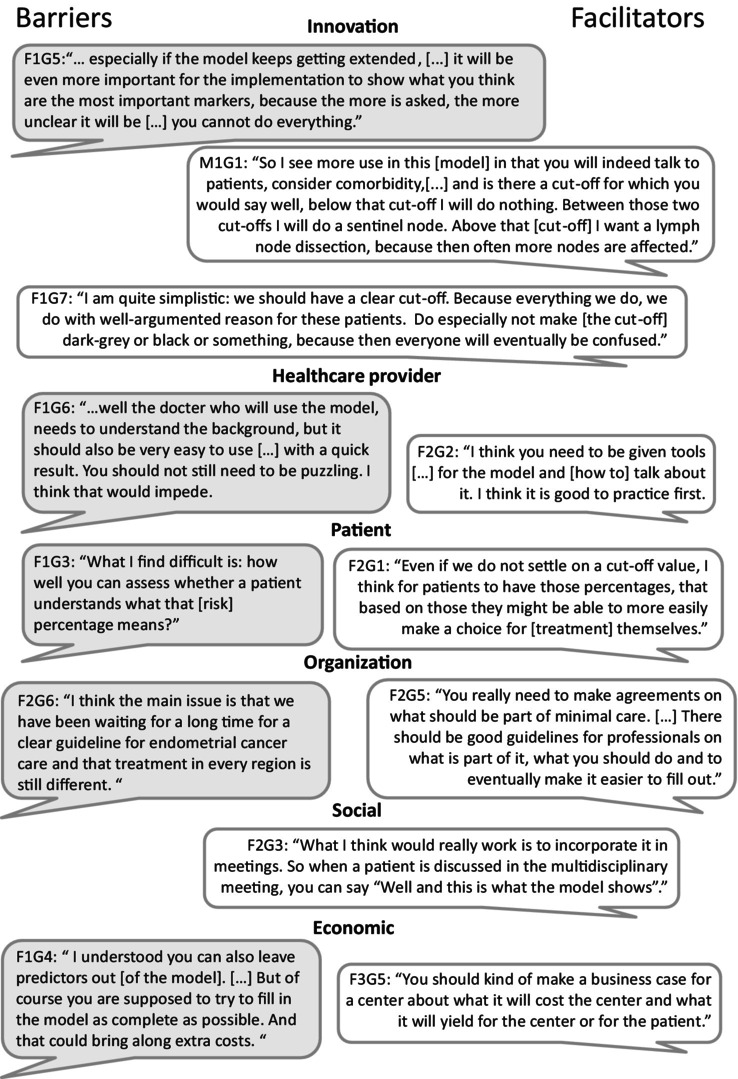
Illustrative quotes.

The mean duration of the focus groups was 67 min with on average five participants per focus group. An overview of barriers and facilitators that could influence use and implementation of the ENDORISK model into clinical practice according to HCP, including the derived recommendations, is shown in Table 2 and described below. Illustrative quotes are demonstrated in Figure 2.

**TABLE 2:** Overview of barriers and facilitators influencing use and implementation of ENDORISK in clinical practice and the derived recommendations.

Themes	Barriers	Facilitators	Recommendations
Relating to the innovation			
Variables in model	Lack of some clinically relevant diagnostic tests in the model	Incorporate diagnostic test variables based on clinical usability needs of HCP and on most recent endometrial cancer guidelines	Start implementation with a risk model that is up to date with diagnostic tests as recommended by the prospective end‐user and the state‐of‐the‐art clinical guidelines to prevent premature updates or changes while users are still getting familiar with the model
	Abundance of variables in the risk model could make it overwhelming to navigate	Provide an overview of minimal variable sets needed for input for sufficient diagnostic accuracy	Define a minimal level of diagnostic model accuracy that is needed for safe clinical use prior to implementation
			Create a clear and concise overview in the model user interface of the minimal variable input needed
			Train HCP in understanding minimal variable input needed to guarantee diagnostic accuracy in clinical practice while maintaining clinical flexibility
Interpretation of risk estimation	Subjective interpretation of model risk estimation of LNM by both HCP and patients could lead to significant differences in treatment within and between hospitals	Create a consensus or guideline with all involved HCP, and include professional associations, on acceptable risk to refrain from surgical LN assessment prior to implementation	Determination of an acceptable risk percentage cutoff for surgical assessment of LN status should include: Validation of the risk model and cutoffs on the prospective target population Whether surgical assessment of LN status is performed by sentinel node procedure and/or lymphadenectomy and the associated morbidity The benefits and risk of possible side effects for a patient from the surgical LN assessment and possible consequent adjuvant treatment Comparing a patients individual risk to what an acceptable risk cutoff would be for the target population as a whole (undertreatment vs. overtreatment)
Relating to individual HCP			
Trust and use of risk estimation	HCP interpret risks differently from patients	HCP should still be able to individualize counseling and treatment while using the model	Ensure HCP understand model input, output and corresponding diagnostic accuracy
			Do not base clinical decisions solely on the risk model, but use ENDORISK as a clinical decision aid
Counseling	HCP do not consistently discuss risk of LNM in *standard care* counseling	Provide sufficient training and education to HCP on how to counsel patients using ENDORISK	Investigate baseline experience and knowledge of HCP in counseling of patients prior to model implementation. Determine the necessary level of training needed accordingly
	HCP are not familiar with counseling patients with exact risk percentages of LNM		
	HCP experience difficulty in explaining “doing nothing” as a treatment option to endometrial cancer patients	Make benefits and disadvantages of treatment options for patients insightful for HCP, provide supportive counseling tools	Training of HCP should incorporate counseling patients about the possible influence of the level of risk of lymph node metastases, as estimated by ENDORISK, on the (adjuvant) therapy decision‐making process
			Develop a decision aid to support HCP in counseling patients about consequences of accepting a low or high risk of LNM as estimated by ENDORISK, including benefits and disadvantages of possible treatment options
Useability	If the risk model takes a long time to use, it would take away valuable consultation time of HCP	If the model is simple to understand and fast to use, it is easier for HCP to incorporate it in daily clinical practice	Make the user interface of the risk model intuitive to prevent the need for extensive training. This should include: An intuitive method of entering variables (e.g., order of variables based on diagnostic process and the minimally needed variables for accuracy) A simple and neutral explanation of how the risk was estimated to leave interpretation for treatment decisions up to HCP and patients For easier interpretation, the risk estimation could be shown in context of the consensus on a general risk cutoff Additional background explanations that can be consulted when needed or when HCP want a deeper understanding of how the model works
Relating to patients			
Influence of model on treatment	Using ENDORISK might lead to reduced treatment for some patients compared to *standard care*, which could be difficult for patients to accept.	Ensure shared‐decision with patients remains leading when implementing ENDORISK in clinical care	Use the model risk estimation as a supporting aid next to a patients' individual preferences, age and phase of life, comorbidity, other available clinical information, and the expertise of the HCP to make decisions about treatment together with patients
	Knowing the exact risk percentage of LNM could lead to patients wanting more extensive treatment, even if not deemed clinically beneficial by HCP		
Provision of information to patients	Explaining the model could be too complex for patients, leading to less informed shared decision‐making	Visualize results of model for patients to increase understanding how the risk is estimated.	Keep initial explanation of the model and the estimated risk simple. Provide educational aids (see “*Relating to HCP,”* theme “Counseling”) to HCP when implementing ENDORISK.
		Model and risk percentage should be easy to understand for patients	Investigate patients' perspectives on preferences for counseling about ENDORISK and risk of LNM. Determine whether patients want to know the exact risk percentage or require other explanations and what other type of information is required
Organizational context			
Availability of diagnostic tests	Not all biomarkers in ENDORISK are yet used by pathologists of all participating hospitals. Not all diagnostic tests might be performed similar in each hospital which could influence model accuracy	Involve pathologists to align performance and scoring of minimally needed biomarkers for ENDORISK	Prior to implementation, an overview should be made in collaboration with all participating pathologists of how biomarkers are stained and scored in each hospital
Hospital capacity	General hospitals might refer more patients if threshold for referral is lowered due to use of ENDORISK. This could lead to capacity problems in referral/academic hospitals and loss of patients in general hospitals	Implementation of ENDORISK could lead to more accurate spread of patients to general versus referral/academic hospitals based on treatment needs	Make agreements with participating hospitals prior to implementation on referrals. Refer “low‐risk” patients back to general hospitals where possible to protect hospital capacity and to ensure patients get treatment as close to their home as possible
Guideline	Implementation without collaborative guidelines between hospitals would lead to treatment differences, which would complicate referrals	Adhering to a collaborative guideline together with all hospitals who will use ENDORISK will allow for implementation entire oncological networks at the same time	Create a guideline together with all hospitals involved and with professional associations for initial implementation of ENDORISK. A guideline should include: The minimal set of variables needed for diagnostic accuracy An overview of specifications that each incorporated diagnostic test should adhere to A consensus on a generally acceptable cutoff that is based on validation of the model on the target population (see domain “*Innovation*,” theme “interpretation of risk estimation”), with suggested consequences for treatment What training users of ENDORISK should have completed How to use, interpret, and how to document use of ENDORISK How to counsel patients when incorporating the risk of LNM as estimated by ENDORISK When to refer patients to referral/academic hospitals for counseling and treatment and when to refer patients back to general hospitals
Sentinel node	Increased tendency toward using sentinel node procedure in all endometrial cancer patients could reduce benefit of using ENDORISK	Incorporating the sentinel node procedure in validations of ENDORISK would facilitate integration	Validate ENDORISK not only in populations of patients who underwent lymphadenectomy, but also sentinel node procedures. ENDORISK could be used to identify candidates for omission of the sentinel node procedure or for omission of additional lymphadenectomy in case of (bi)lateral failure of the sentinel node procedure
Integration in existing systems	Model usage should not be disruptive to clinical process	Incorporate the risk estimation in the electronic patient file for reference in later stages of clinical care	Integrate the user interface or the model itself in existing hospital systems, and in a simple mobile app for HCP. Enable HCP to copy model input and output to a patients' file
Relating to social context			
Attitudinal	Counseling could differ between HCP depending on their own expertise with different surgical treatments and which of those treatments they execute themselves	HCP subspecialized in endometrial cancer care could function as driving force and easily accessible advisor for colleagues	Identify HCP who will be “in the lead” during implementation of ENDORISK in each hospital to motivate correct usage among colleagues
Economic context			
Costs of implementation of ENDORISK in clinical practice	Not all tests incorporated in ENDORISK are part of *standard care* and would be expensive to perform	Reimbursement to hospitals of cost of tests needed for minimal variable set	Educate HCP about minimal variable sets needed for diagnostic accuracy of the risk model to prevent performing additional clinically unnecessary tests. This aids cost‐effectiveness
			Create a business case showing cost and benefit of ENDORISK usage in endometrial cancer care. Involve health insurance for reimbursement to hospitals for use of ENDORISK where possible

### 3.2 Domain: Innovation

#### 3.2.1 Use

According to HCP, ENDORISK could be used in clinical practice by identifying currently under‐ or overtreated patients for surgical LN status assessment in a preoperative setting or in a postoperative setting to assess the benefit of adjuvant therapy. At the time of the study, the SLN procedure was not yet widely implemented. One gynecologist did not see a possible use for ENDORISK with both the increased implementation of the SLN procedure and the use of molecular classification. However, others agreed that incorporating SLN in ENDORISK could facilitate ENDORISK use to determine whether an SLN procedure would be needed or to determine whether lateral lymphadenectomy would be needed if there was (partial) SLN procedure failure.

Most HCP agreed that determining a risk cutoff for LN assessment could be useful in preoperative setting as a starting point for determining the type of surgical treatment. Other patient characteristics such as comorbidity and age would still be important for risk stratification. Some HCP preferred no hard cutoff, as patients' factors might individualize patients “specific cut‐off, while others shared the opinion that it is HCP” responsibility to counsel patients based on an acceptable risk cutoff. Hence, a calculated risk percentage should be seen within of the complete patient assessment in preoperative risk stratification. Factors of influence on deciding on what the cutoff would be, included:

- The need for the identification of benefits to patients and to health care per possible cutoff (amount of false negatives, impact on referral and impact on treatment choices)
- Whether the SLN procedure would be a treatment option: depending on the use of the SLN procedure, some HCP preferred lower cutoff as SLN is associated with reduced surgical‐related morbidity even if this would increase the number of patients allocated to surgical node assessment.
- The benefit of adjuvant treatment on survival: A risk cutoff could also be used to determine which patients might benefit from adjuvant treatment if surgical LN assessment is not possible according to several HCP.
- Undertreatment versus overtreatment per cutoff

#### 3.2.2 Implementation

To implement ENDORISK in clinical practice, most HCP agreed that consensus within an oncological network or preferably, nationally accepted risk for cutoff could facilitate clinical implementation. If each hospital would define its own risk cutoff, both collaboration and referral of patients within oncological networks would be challenging.

When comparing suggested risk cutoffs in EC to other gynecological cancers, such as the 1% cutoff often used in cervical cancer, the large differences between patient populations hampered direct translation. Patients with EC are on average older, with more comorbidity impacting both surgical‐related morbidity as well as personal preferences.

While most HCP agreed upon a cutoff above which surgical node assessment should be assessed, some HCP suggested defining a lower cutoff of 1%–5% under which LN assessment would not be medically beneficial.

**FIGURE 3:**
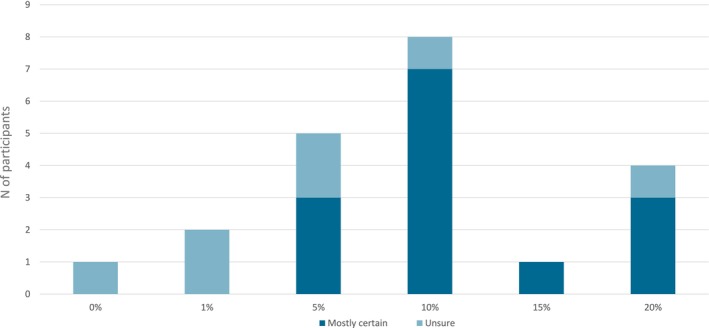
HCP opinions on risk cutoff for lymph node assessment.

Figure 3 shows the distribution of preferred cutoffs among gynecologists as mentioned in the different focus groups. If HCP were uncertain about their preferred cutoff but did mention a number, this was classified as “unsure.”

A barrier to HCP for implementing the model was the lack of certain variables that are recommended for preoperative risk stratification of LNM including: imaging‐based MI, and molecular classification [3]. Although molecular classification was not routinely used by most HCP at the time of focus groups, integration would ensure the model would not need to be updated too frequently once in use and would be more future proof.

Incorporating too many variables was considered to be a barrier, as the model would become too complicated, too time consuming, and too expensive. To facilitate implementation, highlighting the most important variables and showing the accuracy of the calculation (e.g., by showing the confidence interval of the risk estimation) was suggested. This could include showing how important each entered variable would be for the estimated result.

### 3.3 Domain: HCP

#### 3.3.1 Use

Many HCP admitted they do not routinely discuss the estimated risk of LNM with EC patients, partially related to the fact that patients were already referred for surgical LN staging. Other gynecologists and radiation oncologists argued that whether or not to discuss this topic could differ per patient. For other gynecological malignancies like cervical cancer and ovarian cancer, most HCP discuss the risk or use standardized patient information during consultation to explain disadvantages and benefits of surgical treatment. Most HCP discussed the possible disadvantages of LN dissection with patients who have an increased risk of surgical‐related complications. Several gynecologists and a radiation oncologist experienced that patients often want certainty and found it challenging to properly explain the balance between the risk of LNM and patients' specific surgical‐ and adjuvant therapy‐related complications to LN removal.

HCP in most focus groups agreed that a risk estimation model like ENDORISK could aid in providing more insight and accuracy in personalized risk estimation compared to current care.

#### 3.3.2 Implementation

Prior to using ENDORISK in clinical practice, most HCP wanted a training to learn how to use ENDORISK and how to counsel patients with it. This would include understanding not only the outcome, but also how the model calculates the outcome and what variables would be minimally needed for diagnostic accuracy. Furthermore, several HCP wanted training in counseling patients when including ENDORISK in the decision‐making process, including how to interpret the estimated risk, and how to translate this to a simple explanation for patients. Training could include test cases to practice counseling. In addition, some HCP suggested demonstrations of using the model during existing clinical meetings, or specifically to relevant HCP, to incorporate all prospective users in the training.

Gynecologists requested some form of guidance to facilitate the use of ENDORISK in clinical practice, to interpret the calculated risk percentage and tools to counsel patients properly. Examples were an informative card to use during patient consults similar to one they were already familiar with for ovarian cancer, or a clear table to show the benefits and disadvantages of different treatment options and provide uniformity in counseling between clinicians and hospitals.

Several gynecologists agreed that even with a predefined risk cutoff, counseling should not be positive or negative but rather used to explain the benefits and disadvantages of the possible treatment options, including both the surgical treatment and adjuvant therapy.

Another facilitator for implementation according to most HCPs would be the user friendliness of the model. This could be accomplished by creating a user interface in which it is easy to fill in the variable values and see which minimal variables are needed. In addition, making the resulting risk percentage easy to interpret through agreements on a cutoff and visualizing how each variable influences the calculated outcome would help.

### 3.4 Domain: Patient

#### 3.4.1 Use

Most HCP agreed that an acceptable risk of LNM could be different for each patient based on their own preferences, phase of life, comorbidity, and impact of subsequent treatment on their quality of life. In a preoperative setting, the model could be used to show patients their individual risk of LNM as an additional tool besides medical history, medication, current quality of life, and patients' wishes in counseling.

Based on patients' preferences, some HCP questioned whether an exact risk percentage would increase patients' wish to determine surgical LN assessment even with a very low risk where physicians feel it does not outweigh the surgical‐related complications.

#### 3.4.2 Implementation

Most HCP agreed that ENDORISK and the risk percentage should be easy to understand for patients. HCP disagreed on whether or not patients should be able to be told the risk percentage and process behind ENDORISK. It might be too complex and induce anxiety and concern with the patient, while some patients might want to be completely informed and want to understand everything.

Suggestions for explaining a risk of 20% as 1 in 5 (ratio), categorizing the percentages into risk groups based on the cutoff, or some type of visualization of the risk were mentioned. The use of a decision aid was supported by several HCP as a facilitator as well.

### 3.5 Domain: Organizational Context

#### 3.5.1 Use

A risk estimation model could be used on different platforms, preferably on a mobile app, with an option to integrate the risk estimation in the electronic patient file. This would allow HCPs to refer back to the results at a later stage of clinical care, for example, when determining optimal adjuvant treatment postoperatively.

#### 3.5.2 Implementation

ENDORISK incorporates several biomarkers (ER, PR, P53, and L1CAM), which were not routinely performed in all laboratories at the time of the study. HCP agreed that pathologists would need to be part of the implementation process to ensure all necessary biomarkers can be scored in participating hospitals.

Several gynecologists from both referral hospitals expected a barrier if the use of ENDORISK would influence the amount of referred patients for surgical LN removal. This could lead to capacity problems. However, a new risk estimation for patients could also ensure low‐risk patients can be referred back to general hospitals.

Availability of a clear guideline would facilitate ENDORISK implementation, due to current differences within EC care within oncology networks and international guidelines. A guideline should include:

- The minimal set of variables needed as input in ENDORISK for a predefined minimal level of diagnostic accuracy of this risk estimation.
- An overview of specifications that each incorporated diagnostic test should adhere to, ensuring similar interpretation in each hospital.
- The consensus by HCP on a generally acceptable risk cutoff based on validation of the model, preferably on the target population, with suggested consequences for treatment, and how to interpret the individual risk estimation for a patient to tailor personalized treatment.
- What training users of ENDORISK should have completed on usage of ENDORISK and counseling of patients (e.g., following the user‐interface tutorial, test cases for counseling).
- When to refer patients to referral/academic hospitals for counseling and treatment and when to refer patients back to general hospitals.

### 3.6 Domain: Social Context

#### 3.6.1 Use

Several gynecologists identified the difference between care in general and referral centers as a possible barrier for proper counseling of patients. Whether a gynecologist performs surgical assessment of LN could influence patient counseling, which could be harmonized when incorporated in a clinical guideline.

#### 3.6.2 Implementation

Some gynecologists mentioned implementation to be impeded by HCP' personal beliefs in the efficacy of ENDORISK. Others shared the opinion that a dedicated team of one or two HCPs with high knowledge of the model could disseminate clinical use among colleagues.

### 3.7 Domain: Economical and Political Context

#### 3.7.1 Use and Implementation

A change in referral caused by preoperative risk counseling could cause some hospitals to lose part of their patient population with subsequent loss of reimbursements. To facilitate implementation, a business case of costs and benefits for hospitals could give valuable information about the impact of model use on patterns of care, including on referrals, several HCP agreed. In addition, it would be important to know benefits in terms of survival and quality of life. If a business case showed a clear benefit to patients and, to a lesser extent of importance, hospitals, involving healthcare insurance providers could be valuable to facilitate these expenses.

Costs for additional biomarkers were considered a possible barrier. Several HCP mentioned that covering costs for expenses needed to start using the model could facilitate implementation in clinical practice.

## 4 Discussion

A majority of HCP found personalized risk estimation with ENDORISK of beneficial value to preoperative risk stratification of patients based on the estimated risk of LNM. Patient characteristics such as comorbidity, age, medical history, medication, current quality of life, and patients' wishes should remain part of decision‐making as well. Most HCP agreed that ENDORISK could provide more insight and accuracy in risk determination than current care. While several HCP implied they do not always discuss the risk of LNM with their patients, they expected this could change with the use of ENDORISK in clinical practice. Facilitators for use were a consensus on a risk cutoff, showing the accuracy of the calculation, the ability to use the model for both lymphadenectomy and SLN procedure counseling, and an impact analysis to show the influence of model usage on change in treatment and referral of patients. Providing a user‐friendly interface, a cohesive guideline, and training on use and counseling with ENDORISK would facilitate implementation.

### 4.1 Translation of Results to Implementation of ENDORISK in Clinical Practice

For implementation of a risk estimation model such as ENDORISK into clinical practice, incorporation of relevant variables like the molecular subgroups and assessment of MI by MRI or ultrasound are needed to ensure ENDORISK remains future proof after implementation. A study implementing these variables in ENDORISK was recently finished and is awaiting publication.

Based on the available validations of ENDORISK, the increased implementation of the SLN procedure, benefits and risks of morbidity to patients, and preferences of HCP, clinicians might consider omitting a surgical LN assessment below a risk of 5% of LNM as estimated by ENDORISK. A risk cutoff of 10% might be used, above which (additional) lymphadenectomy could be considered. Between 5% and 10%, omitting additional lymphadenectomy could be considered for patients with (bi)lateral failure of the SLN procedure. Based on the initial validations of ENDORISK in a population similar to the intended implementation population, only 4.3% of patients with LNM would be missed at a cutoff of 10% [22].

As HCPs might interpret risks differently from patients, and currently do not consistently discuss the risk of LNM, adequate training of HCPs is required prior to implementation. This should include training HCPs on how to use the ENDORISK user interface. A baseline investigation, prior to implementation, of experience and knowledge of HCPs in counseling patients about the risk of LNM and its consequences for (adjuvant) treatment is needed to tailor the level of training on how to counsel patients with ENDORISK. Visualization tools for the estimated risk and a decision aid could be developed to aid HCPs in counseling patients when ENDORISK is used. A qualitative interview study with endometrial cancer patients is currently ongoing, which will further identify patients' perspectives on preoperative counseling with ENDORISK.

When used in clinical practice, the risk estimation by ENDORISK for LNM should be used as a supportive aid in shared decision‐making, together with consideration of a patient's personal preferences. An additional qualitative study was performed after this focus group study and is in preparation for publication to investigate endometrial cancer patients' perspectives on preoperative risk counseling and the use of ENDORISK. This study will aid in determining how patients want to be involved in counseling, what information is relevant to patients for shared decision‐making on treatment, and how ENDORISK could be incorporated in this.

Additional research and user tests for a user interface of ENDORISK, including an integrated tutorial for the user interface, were already performed by Kleinau et al. Based on the results of this study, which have further improved the usability of ENDORISK [35, 36].

Determination of the performance and interrater variability of the scoring of biomarkers that are incorporated in ENDORISK among pathologists, with results being finalized.

The recommended aspects to include in a guideline for the use of ENDORISK were further discussed with HCP of all participating hospitals for a prospective clinical implementation study of ENDORISK in the Netherlands. This study has incorporated all aforementioned aspects in the protocol, including a recommended general risk cutoff, guidance on training, an e‐learning and educational tool for patients such as a counseling video and decision aid. The study is in the final stages of ethical approval.

Finally, a business case investigating the cost and benefit of ENDORISK usage in clinical practice will be performed to determine the feasibility of further implementation.

### 4.2 Strengths and Limitations

Limited studies exist on the implementation of risk estimation models in gynecologic oncology care, and even fewer specifically for EC. However, the number of models that are being developed for risk estimation of extended disease and outcome in endometrial carcinoma is increasing [37, 38, 39, 40]. Understanding clinical need and barriers and facilitators is vital for a relevant and successful translation of clinical decision (AI) models in research to clinical implementation [34, 41, 42, 43]. To our knowledge, this study is the first to specifically research barriers and facilitators from stakeholders' perspectives for the implementation of such a risk estimation model into clinical practice for endometrial cancer.

Research on barriers and facilitators for implementation of risk estimation models for risk for use in counseling showed similar results to our study: Barriers for implementation were time to complete the model, integration in current IT systems, and lack of knowledge how to use the risk tools; facilitators were sufficient training and clear guidelines [30, 44, 45, 46, 47].

HCP from a wide variation of hospitals and backgrounds were interviewed in a focus group setting for this study. By involving HCP from outside the prospective study regions for the ENDORISK model as well as for the second set of focus groups, diversity in perspectives was maintained as the first set of focus groups was held in specific regions. Due to COVID‐19, a switch was made to a digital setting which might have influenced the quality of discussion. The group size of some focus groups was slightly lower due to some last minute cancellations. As this study aimed to investigate HPC perspectives from entire oncological hospital regions, more focus groups were performed to correct for these cancellations and to ensure data saturation could be reached.

While ENDORISK can be used to estimate the risk of LNM, the role of full lymphadenectomy for staging of EC is associated with increased surgical‐related morbidity [48]. The ultimate goal of determination of nodal status is to tailor adjuvant therapy. With the introduction of the SLN procedure, surgical morbidity is significantly reduced when compared to lymphadenectomy, while showing similar survival rates [13, 48, 49]. This study has shown that even with the clinical implementation of the SLN procedure, ENDORISK could be used for preoperative risk stratification, and determination of whether side‐specific lymphadenectomy is needed when SLN failed. Use of ENDORISK in relation to the SLN procedure will likely lower the preferred cutoff for some HCP or would mean that they preferred multiple cutoffs between no LN assessment, SLN procedure, and lymphadenectomy, which could aid in tailoring the optimal surgical treatment for patients.

## 5 Conclusion

According to HCP, ENDORISK would be a relevant addition to clinical practice for risk estimation and to aid counseling of patients with endometrial cancer. Future implementation of ENDORISK into clinical practice should focus on a user‐friendly interface to aid efficient use by clinicians and informing patients, a cohesive guideline, and training on use of the model and counseling for clinicians. In addition, a business case on the impact of ENDORISK use on the patient population of prospective hospitals could aid motivation and willingness of HCP to use the model. The barriers and facilitators identified in this study were incorporated in the protocol for an upcoming prospective implementation study in which the clinical use of ENDORISK in preoperative counseling will be evaluated.

## Author Contributions

Marike Lombaers: conceptualization, formal analysis, investigation, methodology, visualization, writing – original draft, writing – review and editing. Stephanie Vrede, Casper Reijnen: conceptualization, formal analysis, investigation, methodology, writing – original draft, writing – review and editing. Dorry Boll, Nicole Visser: writing – review and editing. Hanny Pijnenborg: conceptualization, funding acquisition, investigation, methodology, supervision, writing – review and editing. Nicole Ezendam, Rosella Hermens: conceptualization, investigation, methodology, supervision, writing – review and editing.

## Ethics Statement

According to the guidelines of the Dutch Central Committee on Research Involving Human Subjects and the Medical Ethical Committee East Netherlands, this study is exempt from the requirement of ethical approval.

## Consent

All participants provided written informed consent prior to participation in this study.

## Conflicts of Interest

The authors declare no conflicts of interest.

## Supporting information


**Data S1:** cam471103‐sup‐0001‐Supinfo1.docx.

**Data S2:** cam471103‐sup‐0002‐Supinfo2.docx.

## Data Availability

Due to the qualitative nature of this study and to protect study participant privacy, the data of this study are not available for sharing.
