# Supplementary material for: Implementation of a Personalized Risk Model for Lymph Node Metastasis in Endometrial Carcinoma: Healthcare Providers' Perspectives on Use, Barriers, and Facilitators
Source: Cancer Med. 2025 Aug 1;14(15):e71103. doi: 10.1002/cam4.71103 (PMC12314653; doi:10.1002/cam4.71103)
Supplement: Supplementary file 2 — Data S2: cam471103‐sup‐0002‐Supinfo2.docx. [file CAM4-14-e71103-s001.docx]

Interview guide focus groups

# Focus group

Moderator:
Content experts:
Introduction attendees | 5 minutes

Everyone gets the opportunity to introduce themselves with name, function, hospital and years of experience.

Introduction | 15 minutes

The ENDORISK-project was started by Hanny Pijnenborg, within the European consortium ENITEC, and subsidized by an earlier subsidy of the Dutch Cancer Society. Within this project, a prediction model was developed to predict lymph node metastases and outcome in endometrial cancer. We want to bring this model to the consultation room in which it can be supportive for choosing a surgery. For this, different factors play an important role: the chance of lymph node metastases; improving prognosis by adjuvant therapy in case of metastases; side effects of the treatments.

The goal of this meeting is:

1. to hear what you think of the model
2. to research what facilitators or barriers you think are involved in the implementation of a preoperative prediction model to predict lymph node metastases.

It is an open conversation in which you can respond to each other, I am mostly here to ask questions every now and then and to ensure we discuss all important subjects.

If it is all right with you I would like to record the focus group, so we can let it be transcribed and the answers can be analyzed.

Are there any questions before we start?

CR/SV/ML will give a short presentation about the prediction model, and will illustrate it with two practical cases.

## Introduction questions (only in part 1)| 5 minutes

**What do you think of the model?**

**Why do or do you not think the model to be of additional value?**

## Key questions | 60 minutes

**What factors could facilitate or impede use of the prediction model within standard clinical practice?** With this we do not only mean using the model itself, but for example also changes in the health care process (executing a lymph node dissection) or the information provision towards patients.

Level 1. Factors at the level of innovation

**Are there characteristics of the prediction model itself that facilitate or impede implementation?**

**What are barriers and what would reduce these?**

In depth: quality of evidence of the model, attainability of evidence, adaptability to daily practice.

Level 2. Factors at the level of the individual health care professional

**What would, at the level of the individual health care professional, facilitate or impede the use of the model? / What information is needed for proper implementation?**

In depth: knowledge, expectations towards effect on survival, motivation, lifestyle and ability to change.

Level 3. Factors at the level of the patient

**What factors would impede or facilitate the use of the model for patients? / What would facilitate or impede implementation of the model for patients?**

In depth: needs and wishes of patients, communication towards patients, preferences, motivation and behavior.

Level 4. Factors at the level of social context

**To what extent would the use of the model be facilitated or impeded at the level of colleagues within a healthcare team? / To what extent do colleagues within a healthcare team influence implementation of a prediction model?**

In depth: communication and opinions, team processes, proponents and opponents.

Level 5. Factors at the level of the organization

**What would facilitate or impede use of the model at the level of the hospital/department.** Where does the prediction model fit in the healthcare process? **/ Do you foresee problems in implementing such an extra step? What is needed to ensure efficient implementation?**

In depth: priorities, assistance, support of management for implementation, ease/difficulty in implementing change, referral of patients.

Level 6. Factors at the level of finances and regulations (economic context)

**What factors at the level of finances and regulations would facilitate or impede use /implementation of the model?**

In depth: Current guidelines, costs and benefits, quality of care, health care insurers.

Level 7. Other factors

**What other facilitators or barriers might be relevant that have not yet been discussed during this focus group?**

**Participant verification | 15 minutes**

Now that we have discussed all these subjects, we would like to summarize the content discussed in this focus group to verify if we properly collected all your comments and information.

Does anyone have any remarks or subjects that have not yet been discussed?

**Closing | 2 minutes**

I would hereby like to finish up the focus group. Thank you all on behalf of the entire team for your time and valuable input. With your help we can develop a targeted strategy for implementation.
